# Association Between Depression and Medication Adherence Among Post-myocardial Infarction Patients in a South Asian Clinical Setting

**DOI:** 10.7759/cureus.87344

**Published:** 2025-07-05

**Authors:** Samraiz Nafees, Niamat Ali, Abali Wandala, Zeeshan Hussain, Abdullah Ghaith AlShaharli, Khizra Mussadiq, Ramesh Raj Sunar, Maria Ejaz, Aaleen Kamran, Muhammad Rahim Arshad, Zill-e-Rukh Fatima Ameer

**Affiliations:** 1 Internal Medicine, York and Scarborough Teaching Hospitals NHS Foundation Trust, Scarborough, GBR; 2 Internal Medicine, MindWave Research Center, Islamabad, PAK; 3 Internal Medicine, Lahore General Hospital, Lahore, PAK; 4 Interventional Cardiology, Mission Vascular and Vein Institute, Mission, USA; 5 Medicine, Universidad Adventista del Plata, Parana, ARG; 6 Diving Medicine, Armed Forces Hospital, King Abdulaziz Air Base, Dhahran, SAU; 7 Underwater and Hyperbaric Medicine, Pakistan Navy Station Shifa Hospital, Karachi, PAK; 8 Internal Medicine, National University of Science and Technology, Muscat, OMN; 9 Psychiatry and Behavioral Sciences, Nishtar Medical University and Hospital, Multan, PAK; 10 Internal Medicine, Chirayu National Hospital and Medical Institute, Kathmandu, NPL; 11 Internal Medicine, HITEC Institute of Medical Sciences Taxila Cantt, Taxila, PAK; 12 Pathology, Foundation University Medical College, Islamabad, PAK; 13 Internal Medicine, Adam University, Bishkek, KGZ; 14 Internal Medicine, Combined Military Medical College and Hospital, Lahore, PAK

**Keywords:** depression, medication adherence, morisky medication adherence scale, myocardial infarction, patient health questionnaire, south asian population

## Abstract

Background: Medication adherence is vital for successful recovery after a myocardial infarction (MI). Unfortunately, many patients experience depression following an MI, which can negatively affect their ability to stick to prescribed medication plans. It is important to explore how depression influences medication adherence, especially in South Asian clinical environments where cultural factors can significantly impact health outcomes.

Methods: A cross-sectional study was carried out among 385 post-MI patients who attended outpatient clinics in Islamabad, Pakistan. The Patient Health Questionnaire-9 (PHQ-9) was used to measure depression, and the Morisky Medication Adherence Scale (MMAS-8) was used to quantify medication adherence. Statistical tests involved descriptive statistics, normality tests using Shapiro-Wilk, Spearman correlation, Kruskal-Wallis, chi-square, and linear regression. Data were collected between January and May 2025.

Results: There was also a weak, statistically significant positive relation between the PHQ-9 and the MMAS-8 scores (r = 0.124, p < 0.05). In a linear regression model, PHQ-9 scores were significantly predictive of MMAS-8 scores (B = 0.064, p = 0.002), which means that the higher the depressive symptoms, the more likely medication adherence was. Depression scores worsened over time since MI and heavier medication burden, whereas adherence was highest among patients taking moderate numbers of medications (three to five/day). The rates of adherence and psychological distress also differed significantly depending on the treatment type, with the best rates being in angioplasty patients.

Conclusion: This study, unlike the majority of the previous findings, reported a minor positive correlation between self-reported medication adherence and depressive symptoms in post-MI patients. The findings indicate that, in particular, in the cultural backdrop, depressive symptoms can be present alongside more health conscientiousness or planned-out care use. Comorbidities and complexities of treatment should be assessed, and mental health screening and adherence support to medications should be a component of post-MI care strategies.

## Introduction

A myocardial infarction (MI) occurs when there is a blockage in the heart's blood vessels, and the blood flow to that area is reduced. Lack of oxygen leads to damage to the heart muscle [1]. Compliance with medications post-MI is poor, and only approximately 29% of patients uphold good compliance with all classes of medication within a year after release. Nonadherence has been linked to lower clinical outcomes and interacts with demographic factors like age, previous coronary artery bypass grafting (CABG), education level, and marital status [2]. Adherence of ≥80% to guideline-recommended medications following MI is linked with significantly reduced rates of adverse coronary events. Partial or low adherence provides little or no benefit, highlighting the need for sustained full adherence to achieve better outcomes [3].

Overall, drug compliance following MI is usually low and worsens over time. Nonadherence is linked to poorer clinical outcomes, whereas improved adherence tends to go along with enhanced healthy habits and lower readmission or complication risks [4]. Approximately 15-30% of MI survivors report depression in the initial one year. It may result in unfavorable health outcomes and is frequently underdiagnosed because the symptoms overlap. The best course of action is to find the disease in its early stages of development and treat it [5].

Depression impacts numerous patients following MI and contributes to increased mortality rates, in part due to treatment nonadherence and physiological alterations. Enhanced adherence, cardiac rehabilitation, and social support are the most critical measures, whereas the use of antidepressants should be individualized until more data emerge [6]. Depression in the immediate aftermath of MI significantly raises cardiac mortality risk in the following 18 months, particularly among patients with incessant premature ventricular contractions (PVCs). This underscores the importance of early screening and targeted treatment for depression to enhance post-MI outcomes [7].

Adherence to medication in patients with acute myocardial infarction (AMI) was mostly incomplete, and improving adherence was associated with younger age, employment, recent MI, and lower anxiety and depression. Mental health and patient factors should be addressed to enhance adherence and outcomes following AMI [8]. Depression is linked to much worse medication adherence in chronic disease patients, enhancing the chances of nonadherence by roughly 76%. The relationship holds for different diseases, but can be variable based on the measure of adherence. Depression management is essential in enhancing medication adherence as well as improving health outcomes [9]. Depression is associated with medication nonadherence in outpatients who have stable coronary heart disease (CHD). Depressed patients were much more likely to miss or forget medications, and this relation persisted after controlling for other variables. Medication nonadherence can partially account for poorer cardiovascular outcomes among depressed CHD patients [10].

Despite the increasing realization of this relationship all over the world, little research from South Asian clinical practice is available, whereby socio-cultural influences, access to care, and stigmatization can further influence depression as well as medication use [11]. The purpose of this study is to investigate the relationship between depression and drug compliance in post-MI patients within a South Asian clinical setting. Appreciation of this link is important to design focused interventions for enhancing compliance and ultimately for decreasing morbidity and mortality among this at-risk group.

Rationale

Even with the advances in pharmacological treatments and secondary prevention measures, post-MI patients in South Asia remain at high risk for recurrent cardiovascular events, which is the contributing factor to poor adherence to medications [2,12]. Psychological aspects, particularly depression, have also been identified as primary drivers of nonadherence [8]. Cultural beliefs regarding mental well-being, restricted access to psychosocial care, and strained healthcare systems make it even more challenging to manage depression in this part of the world [11]. This study aims to identify a modifiable risk factor that, when addressed, would improve clinical outcomes and decrease healthcare burdens by investigating the relationship between medication adherence and depression in post-MI patients. The study will likely provide geographically relevant information on psychological and cultural factors affecting adherence behavior to medication in post-MI patients, which will justify the inclusion of mental health assessment in cardiac care guidelines in South Asia.

Primary objective

To evaluate the association between depressive symptoms and medication adherence among post-MI patients within a South Asian clinical setting, using the Patient Health Questionnaire-9 (PHQ-9) and the Morisky Medication Adherence Scale (MMAS-8).

Secondary objectives

The research aims to determine the prevalence of depressive symptoms among post-MI patients. Also, the research aims to examine the demographic (age, gender, marital status, economic background) and clinical (comorbidities, prescribed medications, time since MI) factors associated with low adherence levels. Lastly, the research investigated the possibility of depression being an independent factor that contributed to nonadherence despite controlling for other demographic and clinical factors.

## Materials and methods

A cross-sectional study was conducted to look at the connection between depression and taking medicine regularly in people who have had an MI. People in the study group were chosen from post-MI patients visiting outpatient cardiology and internal medicine clinics in Islamabad, Pakistan, and they varied in the severity of their disease and how well they followed their treatment guidelines. The study population was identified through convenience sampling in a tertiary care hospital and its associated primary health centers. This ensured that people in the local population experiencing the outcomes of MI could be part of the study.

To evaluate depression, the PHQ-9 [13] measure was used, and to check medication adherence, the MMAS-8 [14] was applied. Information about other health factors, such as medical history, was recorded via questionnaires and by studying patient documents.

Through this approach, it became possible to review the correlation between depression and drug compliance to learn more about the psychological aspects that can influence a patient’s secondary recovery following an MI in South Asia.

Sample size and technique

The formula for an infinite population was used for determining the sample size for this study because the entire population of post-MI patients within the chosen clinical environment was not known with precision. The formula applied is:

\[n = \frac{Z^2 \cdot p (1 - p)}{d^2}\]

In the formula, *Z* is the z-score at the required confidence level, *p* is the prevalence as estimated, and *d* is the required margin of error. For a 95% confidence level, *Z* = 1.96, and the margin of error (*d*) was assumed to be 0.05. The prevalence (*p*) was assumed to be 40.7% for sample size estimation, as recommended for general prevalence studies when prior data are limited. Based on this value and using the standard sample size calculation formula for prevalence studies, the required sample size was calculated to be 385 [15].

Participants were enrolled through convenience sampling from outpatient cardiology clinics and primary healthcare facilities in Islamabad, Pakistan. This facilitated the recruitment of participants from diverse demographic and clinical backgrounds representative of the heterogeneity of post-MI experiences within the South Asian clinical setting.

The eligibility of study participants was determined based on specific inclusion and exclusion criteria, as detailed in Table 1.

**Table 1:** Inclusion and exclusion criteria for study participants.

Inclusion criteria	Exclusion criteria
Adults aged 18 years and above	History of significant depressive illnesses not including depression (e.g., schizophrenia and bipolar disorder)
History of myocardial infarction (MI) in the past 6-12 months	Undergoing psychiatric treatment for non-depressive conditions
Prescribed at least one secondary prevention medication (e.g., antiplatelets, beta-blockers, statins, angiotensin-converting enzyme inhibitors) after MI	Recognized mental illnesses or brain conditions
Capable of providing informed consent and filling in study questionnaires either alone or with minimal assistance	Serious communication problems
Follow-up care services by a cardiology outpatient facility or a primary health care center affiliated with the hospital	In critical condition or admitted to a hospital with another cardiac event
	Declined to take part in the research

Data collection tools and procedures

During this study, we used structured face-to-face interviews with patients at outpatient cardiology clinics and related primary healthcare centers in Islamabad, Pakistan. Participants were told about the study’s main aim and gave written consent to take part before any data were collected. Surveys involving two tested and validated tools were used to gather information on the primary study subjects: depression and how well patients followed their medication instructions.

Patient Health Questionnaire-9 (PHQ-9)

PHQ-9 is a questionnaire with nine parts that patients fill out by themselves to assess how much depression they are experiencing. Each question is assigned a score from 0 (not at all) to 3 (nearly every day). When the score is 10 or over, it usually means that depression is moderate to severe. The reliability of PHQ-9 is very high, as seen by Cronbach’s alpha of 0.89. Robert L. Spitzer, Janet B.W. Williams, and Kurt Kroenke created it in the year 1999 [13]. PHQ-9 is available for public use and does not require individual permission or licensing. The official version and translations were obtained from the authorized website www.phqscreeners.com.

Morisky Medication Adherence Scale (MMAS-8)

The MMAS-8 is a self-report instrument used to assess how well individuals adhere to their prescribed medication regimens. It classifies the level of adherence as low, medium, or high according to participant rating. The scale covers both nonadherence that is intentional and unintentional, such as when participants forget to take drugs, cease taking medication because of side effects, or stop treatment without the advice of a doctor. Consisting of a mix of yes/no and Likert-type questions, MMAS-8 assesses several behaviors related to adherence. The total MMAS-8 score is between 0 and 8. According to the standard scoring criteria, scores of 8 indicate high adherence, 6 to <8 represent medium adherence, whereas a score of less than 6 implies low adherence. The scale has been found to have excellent reliability with a Cronbach's alpha of 0.83 and was created by Dr. Donald E. Morisky and his colleagues in 1986 [14] and is a copyrighted instrument. In this study, the MMAS-8 was used with formal permission granted by adherence.cc (Certificate Number: 6486-3410-7463-5974-5181) on June 16, 2025.

Apart from these instruments, a concise demographic and clinical information sheet was employed in collecting pertinent data like age, gender, marital status, education, occupation, comorbid conditions, time since MI, and current medications. All information was gathered by trained research assistants to ensure accuracy and consistency. Data collection was conducted from January 2025 to May 2025, and confidentiality and ethical principles were always strictly adhered to.

Statistical analysis

The analysis was conducted with IBM SPSS Statistics version 26 (IBM Corp., Armonk, NY). The frequencies, percentages, means, and standard deviations, which make up the descriptive statistics, were used to summarize the demographic and clinical variables. To evaluate the normality of distribution in continuous variables, the Shapiro-Wilk test was used. Most analyses used non-parametric tests because of the non-normal distribution. A Spearman correlation was performed to analyze the correlation between the PHQ-9 scoring and the MMAS-8 scoring. Comparison between the scores of MMAS-8 at the various categories of time since MI, type of current cardiac treatment, and number of medications prescribed per day was done by the Kruskal-Wallis test. Chi-squared tests were used in determining the point of association among categorical attributes that included the presence of chronic illnesses, type of treatment regimens, the number of drugs, and duration after MI. Also, a linear regression was performed to prove that PHQ-9 scores might be used to predict MMAS-8 scores, and a scatter plot of regression standardized residuals was plotted to verify the assumptions on linearity and homoscedasticity. All statistical tests were carried out at a significance level of p < 0.05.

Ethical considerations

The Institutional Review Board of MindWave Research Center, Islamabad, Pakistan (IRB-2025-0026) provided ethical approval for this study. Everyone included in the study was well-informed about what would happen, the study's objectives, potential risks, and the benefits that might be expected. All individuals involved in the study provided written consent prior to participating. Confidentiality and anonymity of responses were guaranteed to participants, and data were coded to maintain the confidentiality of individual identities. They were also notified of their freedom to withdraw from the study at any time without affecting their ongoing medical care. All data were stored securely and used solely for research purposes by institutional and ethical standards.

## Results

Table 2 presents the demographic characteristics of the 385 participants. Regarding age distribution, 70 participants (18%) were aged 18-30 years, 72 (18%) were 31-40 years, 87 (23%) were 41-50 years, 119 (31%) were 51-60 years, and 37 (10%) were aged 61-70 years. On a gender basis, 70 participants (18%) identified as male, 207 (54%) identified as female, and 108 (28%) preferred not to state their gender. The marital status indicated that 51 (13%) were single, 141 (37%) were married, 160 (41%) were widowed, and 33 (9%) were divorced or separated. In educational terms, 24 (6%) had no formal education, 78 (20%) had a primary school level education, 143 (37%) had a secondary school education, 81 (21%) had a higher secondary education, 42 (11%) had a bachelor's degree or higher, and 17 (4%) held postgraduate qualifications.

**Table 2:** Demographic characteristics of participants (N = 385). PCI: percutaneous coronary intervention; TIA: transient ischemic attack; COPD: chronic obstructive pulmonary disease; f: frequency; %: percentage.

Variable	f	%
Age	-	-
18-30 years	70	18
31-40 years	72	18
41-50 years	87	23
51-60 years	119	31
61-70 years	37	10
Gender	-	-
Male	70	18
Female	207	54
Prefer not to say	108	28
Marital status	-	-
Single	51	13
Married	141	37
Widowed	160	41
Divorced/separated	33	9
Educational level	-	-
No formal education	24	6
Primary school	78	20
Secondary school	143	37
Higher secondary	81	21
Bachelor's degree/graduate	42	11
Postgraduate	17	4
Employment status	-	-
Student	25	6
Employed	53	14
Self-employed	137	36
Unemployed	111	29
Retired	59	15
Type of myocardial infarction	-	-
ST-elevation myocardial infarction (STEMI)	75	19
Non-ST-elevation myocardial infarction (NSTEMI)	180	47
Unsure/not informed	130	34
Time since the last myocardial infarction	-	-
Less than 1 month ago	37	10
1-3 months ago	95	25
3-6 months ago	161	42
6-12 months ago	79	20
More than 1 year ago	13	3
Current cardiac treatment	-	-
Medications only	35	9
Angioplasty with stent (PCI)	142	37
Coronary artery bypass grafting (CABG)	142	37
Cardiac rehabilitation program	40	10
Other	26	7
Number of prescribed medications per day	-	-
1-2 medications	70	18
3-5 medications	133	34
6-8 medications	131	34
More than eight medications	51	13
Presence of other chronic illness	-	-
Hypertension	30	8
Diabetes mellitus	62	16
Chronic kidney disease	139	36
Stroke or TIA	101	26
COPD or asthma	41	10
None	12	3
Smoking status	-	-
Current smoker	72	19
Former smoker	165	43
Never smoked	148	38

Concerning employment status, 25 (6%) were students, 53 (14%) were workers, 137 (36%) were business owners, 111 (29%) were unemployed, and 59 (15%) were retired. Among the MI type, 75 (19%) had ST-elevation MI (STEMI), 180 (47%) had non-ST-elevation MI (NSTEMI), and 130 (34%) did not know or were not informed about it. Duration after the previous MI was different: 37 people (10%) had it within the previous month, 95 (25%) between one and three months, 161 (42%) between three and six months, 79 (20%) between six and 12 months, and 13 (3%) over one year.

Regarding current cardiac treatment, 35 subjects (9%) were on medication alone, 142 (37%) received angioplasty with stent (percutaneous coronary intervention, PCI), 142 (37%) underwent coronary artery bypass grafting (CABG), 40 (10%) participated in a cardiac rehabilitation program, and 26 (7%) mentioned other treatments. The daily number of prescribed medications revealed that 70 subjects (18%) received one to two medications, 133 (34%) received three to five medications, 131 (34%) received six to eight medications, and 51 (13%) received more than eight medications per day.

Concerning other chronic diseases, 30 (8%) participants had hypertension, 62 (16%) had diabetes mellitus, 139 (36%) had chronic kidney disease, 101 (26%) had a history of stroke or transient ischemic attack (TIA), 41 (10%) had chronic obstructive pulmonary disease (COPD) or asthma, and 12 (3%) had no other chronic diseases. Lastly, in smoking status, 72 (19%) of the participants were current smokers, 165 (43%) were former smokers, and 148 (38%) were never smokers.

Table 3 shows that the Kolmogorov-Smirnov and Shapiro-Wilk tests were used to evaluate the normal distribution of the PHQ and the MMAS for the data. Statistically, both tests showed significant results based on each of the variables, with a p-value that is below 0.001. In particular, the PHQ had a Kolmogorov-Smirnov statistic value of 0.111 and a Shapiro-Wilk statistic value of 0.984. In contrast, the MMAS had a Kolmogorov-Smirnov statistic value of 0.199 and a Shapiro-Wilk statistic value of 0.941. Since all the p-values are smaller than 0.05, the rule of normality is not met in both variables. The findings imply that the data are not normally distributed, and, therefore, the non-parametric statistical tests must be used in future analyses.

**Table 3:** Normality test results for PHQ-9 and MMAS-8 using Kolmogorov–Smirnov and Shapiro–Wilk tests. df: degrees of freedom; Kolmogorov–Smirnov and Shapiro–Wilk tests were used to assess normality. ** = p < 0.001 considered statistically significant.

Variable	Kolmogorov-Smirnov			Shapiro-Wilk		
	Statistic	df	p	Statistic	df	p
Patient Health Questionnaire	0.111	385	<0.001^**^	0.984	385	<0.001^**^
Morisky Medication Adherence Scale	0.199	385	<0.001^**^	0.941	385	<0.001^**^

Table 4 displays the Spearman correlation of depression levels between the PHQ and the MMAS. There was a statistically significant positive correlation between the two variables (r = 0.124, p < 0.05) that showed that a greater severity of depressive symptoms was linked to some improvement in the levels of medication adherence in the sample. The correlation coefficient is weak despite the unexpected direction, which is likely due to contextual/cultural factors influencing patient behavior in this population.

**Table 4:** Intercorrelations between the study variables. * p < 0.05 is considered significant; correlation= Spearman’s correlation.

Variable	Patient Health Questionnaire	Morisky Medication Adherence Scale
Patient Health Questionnaire	-	0.124^*^
Morisky Medication Adherence Scale	0.124^*^	-

Table 5 indicates that both depression (PHQ-9) and medication adherence (MMAS-8) scores were shown to have significant differences between groups classified according to the time since the last MI. There was a significant difference in depression scores by time since MI (x2 = 15.39, p = 0.004), whereby a larger mean rank was found among the patients whose MI was more than one year ago or within six to 12 months, indicating a higher level of depressive symptoms in these groups. There were also significant findings between medication adherence scores and time categories (x2 = 13.98, p = 0.007), with adherence being highest in patients one to three months post-MI, with increasing decline in adherence in patients with more distant MI. Such findings reveal that the extent of psychological distress, as well as the degree of adherence behavior, changed throughout the recovery period, which may have necessitated varying clinical approaches during the various recovery phases after an MI.

**Table 5:** Kruskal–Wallis test results for PHQ-9 and MMAS-8 scores across time since last myocardial infarction. n: number of participants; x2: effect size; df: degree of freedom. * = p < 0.05, ** = p < 0.01 consider significant; non-parametric = Kruskal-Wallis test.

Time since the last myocardial infarction	n	Patient Health Questionnaire-9 (PHQ-9), mean rank	Morisky Medication Adherence Scale (MMAS-8), mean rank
Less than 1 month ago	37	201.39	162.30
1–3 months ago	95	162.42	220.45
3–6 months ago	161	191.07	200.62
6–12 months ago	79	221.56	178.10
More than 1 year ago	13	242.88	140.15
Test statistics	-	-	-
Variables	x^2^	df	p
Patient Health Questionnaire	15.39	4	0.004^**^
Morisky Medication Adherence Scale	13.98	4	0.007^**^

Table 6 indicates statistically significant differences between the scores of both depression (PHQ-9) and adherence to medication (MMAS-8) concerning the forms of cardiac treatment. It was found that depression levels differed significantly by treatment group (X2 = 16.46, p = 0.002); there were the highest depression scores among patients in the treatment group of "Other" and patients during cardiac rehabilitation or CABG. The group that received angioplasty (PCI) had the lowest scores of depression instead. The medication adherence also differed considerably (chi-square = 14.23, p = 0.006), where patients with PCI were the most adherent, and patients in the groups of "other" and "medications only", correspondingly, were the least adherent. These results indicate that the nature of cardiac intervention has both psychological and analytical well-being, and the related behaviors in accordance with invasive interventions, such as PCI, can be correlated with a higher level of adherence and lower distress than less invasive or unspecified procedures.

**Table 6:** Kruskal–Wallis test comparing PHQ-9 and MMAS-8 scores across current cardiac treatment. PCI: percutaneous coronary intervention; n: number of participants; x2: effect size; df: degree of freedom. * = p < 0.05, ** = p < 0.01 consider significant; non-parametric = Kruskal-Wallis test.

Current cardiac treatment	n	Patient Health Questionnaire-9 (PHQ-9), mean rank	Morisky Medication Adherence Scale (MMAS-8), mean rank
Medications only	35	181.73	155.62
Angioplasty with stent (PCI)	142	166.48	220.78
Coronary artery bypass grafting (CABG)	142	208.29	192.34
Cardiac rehabilitation program	40	216.59	170.41
Other	26	233.23	130.25
Test statistics	-	-	-
Variables	x^2^	df	p
Patient Health Questionnaire	16.46	4	0.002^**^
Morisky Medication Adherence Scale	14.23	4	0.006^**^

Table 7 indicates that there were notable differences in the levels of depression (PHQ-9) and medication adherence (MMAS-8) based on the groups formed around the number of drugs prescribed per day. Patients who were using more than eight medications scored the highest on depression (mean rank = 238.30), whereas the lowest depression rank was observed in those taking fewer medications. This implies that the higher the medication load, the more psychological distress there is. Regarding adherence, in patients with three to five medicines per day, it was the highest (mean rank = 212.06), and the lowest in those taking very few and many medications. The results show that under- and over-prescription could be related to lower adherence, and polypharmacy could add a burden to emotions.

**Table 7:** Kruskal–Wallis test comparing PHQ-9 and MMAS-8 scores across the number of prescribed medications per day. n: number of participants; x2: effect size; df: degree of freedom. * = p < 0.05, ** = p < 0.01 consider significant; non-parametric = Kruskal-Wallis test.

Number of prescribed medications per day	n	Patient Health Questionnaire-9 (PHQ-9), mean rank	Morisky Medication Adherence Scale (MMAS-8), mean rank
1-2 medications	70	177.47	164.31
3-5 medications	133	180.17	212.06
6-8 medications	131	196.69	192.97
More than 8 medications	51	238.30	182.74
Test statistics	-	-	-
Variables	x^2^	df	p
Patient Health Questionnaire	11.84	-	0.008^**^
Morisky Medication Adherence Scale	9.64	-	0.022^*^

Table 8 presents the findings from a linear regression analysis that investigates how well the PHQ-9 scores can predict medication adherence, as assessed by the MMAS-8. It was found that there is a statistically significant link between the PHQ-9 and MMAS-8 scores (p = 0.002). The unstandardized regression coefficient (B) of the PHQ-9 is 0.064, and its 95% confidence interval is 0.023-0.104, with a standardized beta (β) value of 0.156. This means that for every one-point increase in the PHQ-9 score, the MMAS-8 adherence score goes up by 0.064 points. This suggests a small yet meaningful positive link between psychological symptoms and medication adherence. In simpler terms, higher levels of psychological distress were linked to slightly better medication adherence in this sample.

**Table 8:** Linear regression analysis predicting the Morisky Medication Adherence Scale (MMAS-8) scores using the Patient Health Questionnaire (PHQ-9). B: coefficient; S.E.: standard error; β: standardized coefficient; LL: lower limit; UL: upper limit; CI: confidence interval. ** = p < 0.01 considered significant.

Variable	B	95% CI, LL	95% CI, UL	S.E.	β	P
Constant	10.871	9.902	11.84	0.493	-	<0.001^**^
Patient Health Questionnaire	0.064	0.023	0.104	0.021	0.156	0.002^**^

**Figure 1:**
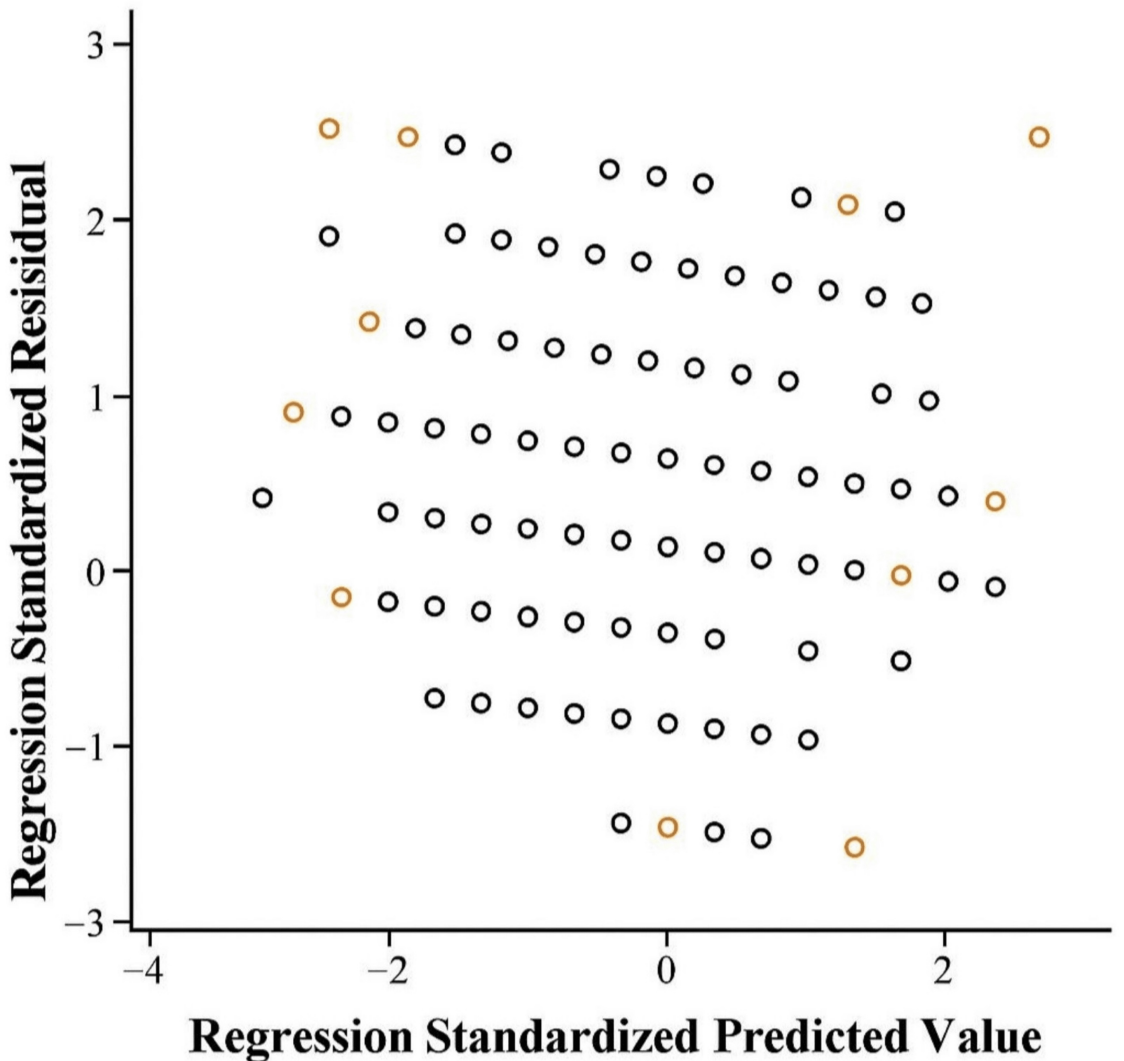
Scatter plot of regression standardized residuals for the Morisky Medication Adherence Scale.

Figure 1 presents the scatter plot of standardized residuals from the linear regression model. The distribution appears randomly scattered around the horizontal axis, indicating no clear patterns or heteroscedasticity. This suggests that key assumptions of linear regression, including linearity, homoscedasticity, and normality of residuals, have been met. Therefore, the regression findings, which show a significant relationship between PHQ-9 and MMAS-8 scores, are statistically valid and reliable.

Table 9 dives into the connection between the time elapsed since the last MI and two key factors: the current cardiac treatment being administered and the daily count of prescribed medications. The chi-square test showed a significant link between the time since MI and the type of cardiac treatment currently in use (χ² = 61.5, p < 0.001). For patients who had an MI less than a month ago (n = 37), four were on medications alone, seven had angioplasty with a stent, 11 underwent CABG, eight were participating in a cardiac rehabilitation program, and seven reported other treatment options. A greater number of patients who experienced an MI between one and three months (n = 95) and three and six months ago (n = 161) received more intensive treatments like angioplasty (n = 48 and n = 62, respectively) and CABG (n = 28 and n = 63, respectively). As the time since the MI stretched beyond six months, the treatment approach shifted more toward medications and other forms of care, indicating a possible transition from acute to long-term management strategies.

**Table 9:** Descriptive statistics of demographic variables (time since last myocardial infarction, current cardiac treatment, number of prescribed medications per day). f: frequency; %: percentage; df: degree of freedom; p: level of significance. P-values were calculated using the chi-square test; the significance level is set at p < 0.05; ** = p < 0.01 is considered significant.

Variables	f	Current cardiac treatment					p	df	x2	Number of prescribed medications per day				p	df	x2
		Medications only	Angioplasty with stent	Coronary artery bypass surgery	Cardiac rehabilitation program	Other				1-2 medications	3-5 medications	6-8 medications	More than 8 medications			
Time since the last myocardial infarction	-	-	-	-	-	-	<0.001**	16	61.5	-	-	-	-	<0.001**	12	35
Less than 1 month ago	37	4	7	11	8	7	-	-	-	7	8	9	13	-	-	-
1-3 months ago	95	11	48	28	3	5	-	-	-	24	34	28	9	-	-	-
3-6 months ago	161	9	62	63	19	8	-	-	-	22	69	54	16	-	-	-
6-12 months ago	79	5	23	38	9	4	-	-	-	16	20	33	10	-	-	-
More than 1 year ago	13	6	2	2	1	2	-	-	-	1	2	7	3	-	-	-

Similarly, a notable association was also observed between the time since MI and the number of medications prescribed daily (χ² = 35.0, p < 0.001). Among those who had an MI less than a month ago, most were prescribed more than eight medications each day (n = 13), while others were on six to eight (n = 9), three to five (n = 8), or just one to two medications (n = 7). Patients in the three to six months post-MI group (n = 161) were more frequently prescribed three to five medications (n = 69) and six to eight medications (n = 54), suggesting a gradual reduction in the intensity of pharmacological management over time. Those who were more than a year post-MI (n = 13) mostly fell into the lower medication categories, indicating that medication regimens tend to become more streamlined as recovery continues. These findings collectively underscore how both the type of treatment and the medication load are significantly shaped by the time elapsed since the cardiac event.

Table 10 shows the link between having other chronic illnesses and two important factors: the current cardiac treatment patients are receiving and the number of medications they take each day, all analyzed through chi-square tests. A noteworthy relationship emerged between the presence of comorbid chronic illnesses and the type of cardiac treatment being administered (χ² = 39.6, p = 0.006). For instance, among patients with hypertension (n = 30), the majority underwent either coronary artery bypass surgery (n = 13) or participated in a cardiac rehabilitation program (n = 5), while a smaller number were treated solely with medications (n = 2) or received angioplasty (n = 6). In a similar vein, patients with diabetes mellitus (n = 62) predominantly had angioplasty (n = 26) or bypass surgery (n = 21), hinting at a trend toward more aggressive treatment options for those with diabetes. This pattern was also evident in patients with chronic kidney disease (CKD) (n = 139) and those who had experienced a stroke or TIA (n = 101), where most had undergone invasive procedures like angioplasty or CABG, suggesting a higher cardiac risk profile.

**Table 10:** Descriptive statistics of demographic variables (presence of other chronic illness, current cardiac treatment, number of prescribed medications per day). TIA: transient ischemic attack; COPD: chronic obstructive pulmonary disease; f: frequency; %: percentage; df: degree of freedom; p: level of significance. P-values were calculated using the chi-square test; the significance level is set at p < 0.05; ** = p < 0.01 is considered significant.

Variables	f	Current cardiac treatment					p	df	x^2^	Number of prescribed medications per day				p	df	x^2^
		Medications only	Angioplasty with stent	Coronary artery bypass surgery	Cardiac rehabilitation program	Other				1-2 medications	3-5 medications	6-8 medications	More than 8 medications			
Presence of other chronic illnesses	-	-	-	-	-	-	0.006^**^	20	39.6	-	-	-	-	<0.001^**^	15	46.9
Hypertension	30	2	6	13	5	4	-		-	2	6	8	14	-		-
Diabetes mellitus	62	9	26	21	3	3	-		-	16	26	14	6	-		-
Chronic kidney disease	139	11	60	51	13	4	-		-	21	50	55	13	-		-
Stroke or TIA	101	9	34	39	11	8	-		-	16	36	37	12	-		-
COPD or asthma	41	1	15	17	4	4	-		-	10	12	13	6	-		-
None	12	3	1	1	4	3	-		-	5	3	4	0	-		-

Additionally, a strong association was identified between the presence of chronic illnesses and the number of medications prescribed daily (χ² = 46.9, p < 0.001). Patients with hypertension were more likely to be on more than eight medications each day (n = 14), while those with CKD showed a balanced distribution between taking three to five medications (n = 50) and six to eight medications (n = 55), highlighting the complexities involved in managing these conditions. Patients with COPD or asthma (n = 41) and those with stroke/TIA also had a significant presence in the higher medication categories, with many taking six to eight or more medications daily. Interestingly, participants without any chronic illnesses (n = 12) had a lighter medication load overall, with five on just one to two medications and none exceeding eight medications per day. These findings highlight just how much comorbid chronic illnesses can affect the intensity of cardiac treatment and the daily medication routines for patients who have had an MI. People dealing with multiple chronic conditions often find themselves needing more aggressive treatment and a more complicated medication plan, which emphasizes the importance of providing thorough, personalized care for this group.

## Discussion

This study investigated the relationship between depression and medication adherence in post-MI patients within a South Asian clinical setting. Unexpectedly, our results indicate there is a weak positive relationship between depressive symptoms and medication adherence. This contrasts with most of the existing literature, which typically characterizes depression as a barrier to medication adherence [16]. One possible explanation is that patients experiencing psychological distress could also be health-anxious or reliant upon highly structured routines, hence enhancing their medication adherence. The other possibility is that such patients are more often interacting with healthcare services due to their mental health symptoms, which improves their adherence indirectly through more oversight. Reverse causality is also possible, as those who adhere better may interact more frequently with healthcare professionals and, therefore, have a greater chance of identifying or revealing depressive symptoms. Moreover, this result could correspond to the processes discussed in the health belief model, as people with a more perceived susceptibility or severity of illness are more inclined to follow prescribed treatment even when they experience psychological distress.

Our results showed an increase in depression scores over time since MI, which suggested that the symptoms of depression were increasing with time. This aligns with previous studies that revealed that chronic illness may increase the threat of depression. The mentioned study also highlights the influence of comorbid factors and social conditions on psychological outcomes [17]. The present finding is consistent with previous studies, which indicated that high medication adherence after MI leads to better clinical outcomes. Just as our study underscores adherence over time, the present research reaffirms that partial adherence gives little benefit and must be treated in conjunction with total adherence to all medications for optimal recovery [3].

The results of our study confirm some previous studies that many cardiac rehabilitation patients report continuing to experience substantial psychological distress. In our research, the most significant scores were recorded by participants undergoing cardiac rehabilitation and other less-defined treatments that demonstrated persistent emotional difficulties. These findings emphasize the urgent necessity of cardiac rehabilitation programs to incorporate more efficient management of physical recuperation and emotional support [18]. Our study results indicated low medication compliance among cardiac rehabilitation patients compared to other types of treatment. However, earlier studies have also found better compliance among patients undergoing cardiac rehabilitation. Such a contrast can be attributed to dissimilarities in the population studied, healthcare environments, or how adherence is measured [19].

In our research, we discovered a link between the number of medications a person takes and an increase in psychological distress. This finding is consistent with another study that focused on older adults dealing with chronic illnesses. That study also highlighted how taking more medications can lead to greater psychological distress, with emotional self-efficacy playing a partial role in mediating the connection between medication use and distress [20]. We found that patients with a moderate number of medications (three to five per day) demonstrated the best adherence rates, whereas those who took few medications showed even lower adherence, and the ones who took more medications demonstrated even lower adherence. This U-shaped pattern indicates that under-treatment and complex polypharmacy might create problems in the consistent pattern of taking medication. These findings are consistent with the findings of earlier research suggesting that complex dosing procedures and an increased number of pills frequently result in loss of adherence, particularly in cases when patients are overburdened with the number of pills or the time of taking them [21]. Furthermore, our observation that patients who take less medication were less adherent is consistent with earlier studies, which indicated that patients who had low levels of adherence to cardiovascular care had taken much less medication and fewer times a day in comparison to the high level of adherence [22]. The reasons behind such an association were not investigated, but it is also plausible that patients on less complex regimens might not treat their illness any more seriously or might not have the systematic routine that affords them a robust way into behavioral adherence; again, a field that should be further investigated.

In contrast to the existing evidence, our regression showed a weak but statistically significant positive relationship between depressive symptoms and medication adherence (B = 0.064, p = 0.002). It implies that, in our sample, greater psychological distress was associated with a modestly improved self-reported adherence. Also, the patients who were prescribed more medications indicated slightly greater psychological distress. Though these findings should be viewed with caution, they suggest that the pattern of psychological influences on adherence is complex and points to a possible role of structured support in care after MI [23].

Results from our research are like those in earlier studies: the amount of time since an MI is significant when choosing what treatments and medications to use. As was seen before, patients who were recently diagnosed with MI generally received more intensive care, such as having angioplasty or CABG. However, as individuals advance in their recovery, their care moves toward using more medication. This illustrates a clear move from immediate care to strategies aimed at long-term management [24]. Our research supports earlier studies that indicate medication adherence tends to drop over time following an MI. Just like in those studies, we found that as more time passes since the MI, patients tend to take fewer medications, and their adherence levels fall. Additionally, the study points out that cardiac rehabilitation can play a significant role in boosting medication adherence, which resonates with our observations that medication plans become more streamlined during the long-term management phase [25].

Our research supports the idea that having other health issues can greatly affect the kind of heart treatment and medications a patient receives. Just like with heart failure patients, those dealing with additional chronic conditions such as high blood pressure and diabetes often need more aggressive treatments and intricate medication plans. This highlights the importance of tailoring care to everyone [26]. Our research aligns with a study that highlights how crucial it is to consider the complexity of medication regimens for patients dealing with chronic illnesses. Just like people with several health problems, the study found that complicated medication plans often result from frequent use of different types of drugs. Multi-drug regimens are often complex and may be a source of unintentional nonadherence. Optimal health outcomes might also be optimized by simplifying treatment regimens of patients with multiple chronic diseases to encourage drug compliance [27].

Although our results provide an in-depth picture, they should be regarded cautiously since they are framed by the cross-sectional nature of the study and reporting bias concerns, which restrict the extent of causal connection and can affect the resulting association.

Limitations

The cross-sectional nature of our study constrains inference of any causal relationship and also incapacitates the ability to evaluate any potential change in the levels of depressive symptoms and how the sense of medication adherence may change with time. It was also single-center research that recruited only one area in South Asia; thus, the external validity and broad applicability of the research findings to other regions or healthcare contexts are restricted. Also, convenience sampling was used in the study, which can introduce selection bias and reduce the generalizability of findings outside of the sampled clinical population. Moreover, the percentage of women and widows in our sample is relatively high; this can also cause sampling bias and may also change the representativeness of the outcomes. Depressive symptoms and medication adherence were both assessed through the self-report methods (PHQ-9 and MMAS-8); both types of measures may be biased due to recall and social desirability. The patients might have reported their compliance to be higher than it might have been or their psychological distress to be lower so that they could portray themselves in a positive way. Also, the PHQ-9 is not a clinical psychiatric diagnosis tool, only a screening tool. Another possibility to keep in mind is reverse causality: when people are more treatment adherent, they may be more self-conscious and more prone to reporting depressive symptoms. Lastly, our research did not gather intensive data about social support, financial pressures, and access to healthcare, which are likely to play an important role in defining mental health and adherence to medication.

Future directions

Future studies are recommended to use longitudinal designs to determine the long-term correlation between medication adherence and depression in post-MI patients. Future studies need to be more representative in their samples, have a more balanced distribution of gender and marital status, and be conducted across multiple centers to increase generalizability. Self-report constructs could be improved by combining clinician-administered diagnostic interviews of depression as well as self-report measures. Additionally, looking at the aspect of interventions, like the use of psychological counseling, antidepressants, or patient adherence-based support programs, would be an eye-opening experience in determining the outcome of such patients. Lastly, adherence is a behavior that is likely to be determined highly by contextual factors such as socioeconomic status, social support of the patient, and access to health care, and these parameters should be included in the data used in future studies.

## Conclusions

Within the limits of a cross-sectional, self-reported design, our study found a statistically significant but weak positive association between depressive symptoms and medication adherence in post-MI patients. This is contrary to most previous research, which typically reports a negative relationship between these variables. Moreover, depressive symptoms were also identified as being more intense as time passed since the occurrence of MI, and were more intense in those patients who were prescribed more medications. These results indicate the significance of considering mental health when working in cardiac care and the necessity of further longitudinal and culturally informed studies that could help to determine the way psychological and treatment-related experiences interact in various populations. Both physical and emotional health necessities should be considered to enhance long-term recuperation through post-MI care.
